# Supplementary figures and images for: Aberrant cortical development is driven by impaired cell cycle and translational control in a DDX3X syndrome model
Source: eLife. 2022 Jun 28;11:e78203. doi: 10.7554/eLife.78203 (PMC9239684; doi:10.7554/eLife.78203)

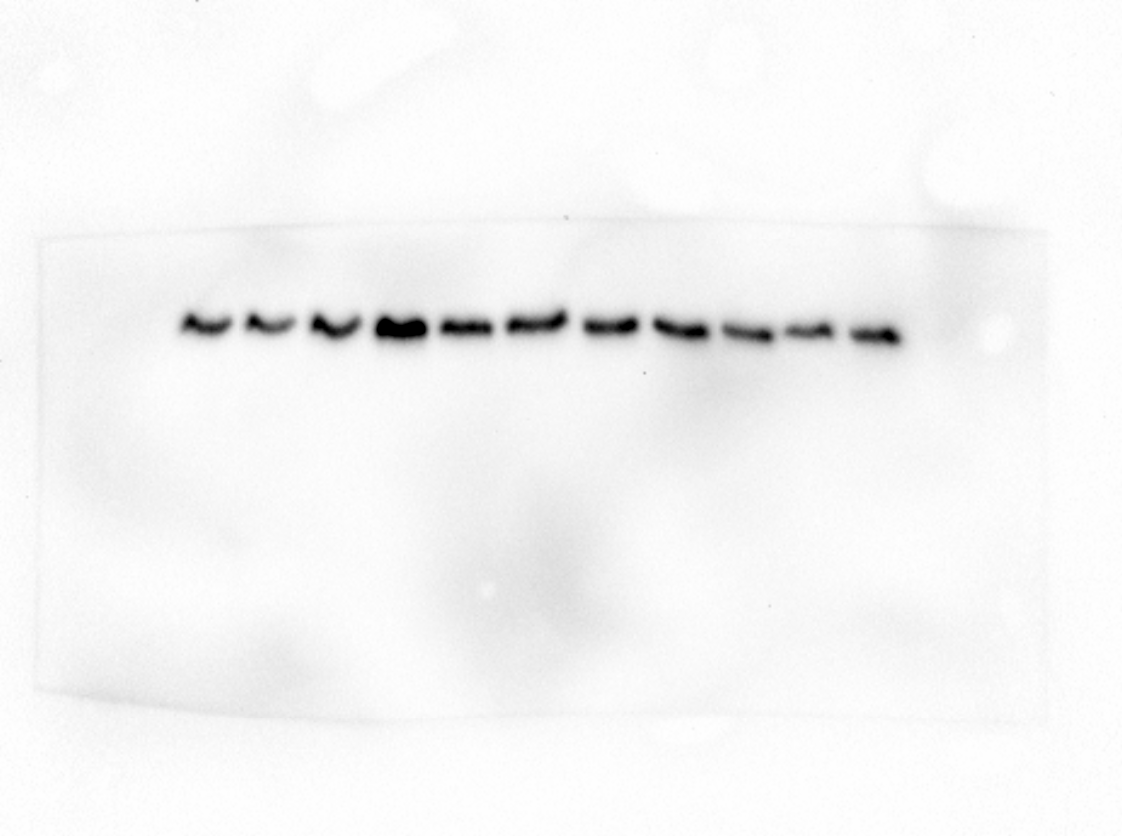

Supplement: Figure 1—figure supplement 1—source data 1. [file elife-78203-fig1-figsupp1-data1.zip › Figure 1_figure supplement 1_A_bactin.tif]

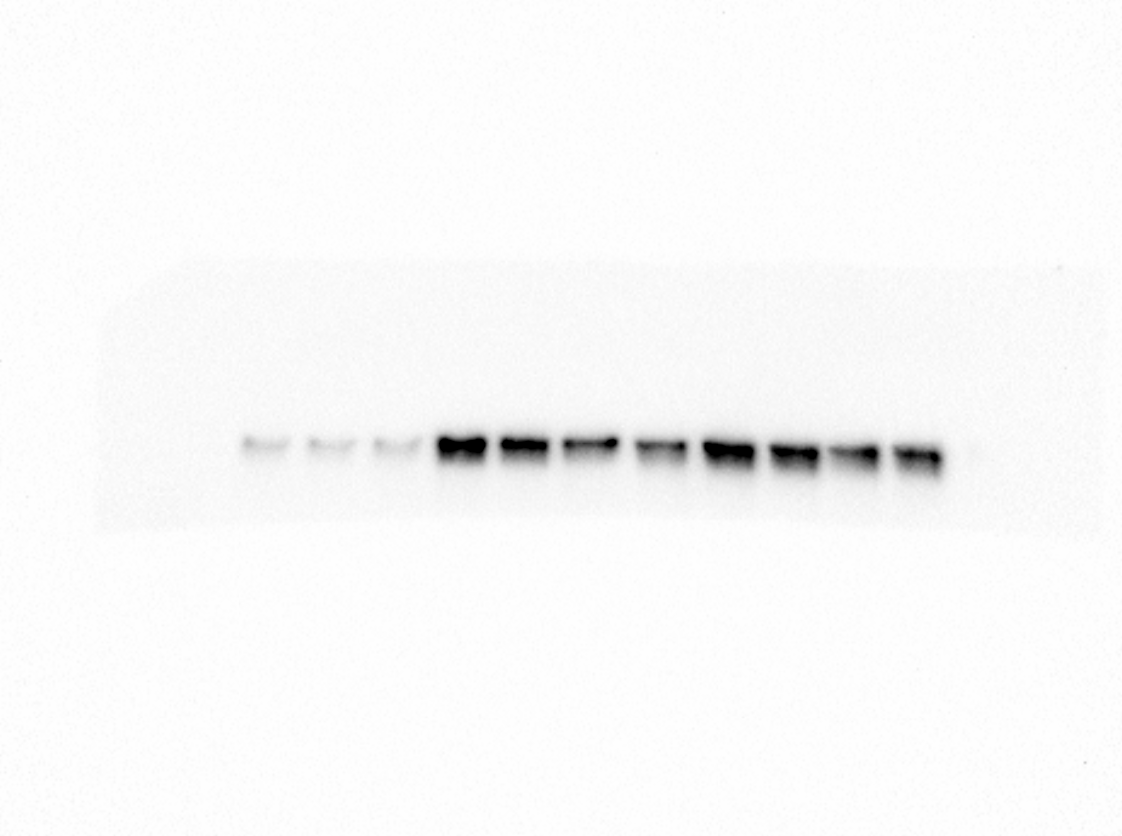

Supplement: Figure 1—figure supplement 1—source data 1. [file elife-78203-fig1-figsupp1-data1.zip › Figure 1_figure supplement 1_A_Ddx3x.tif]

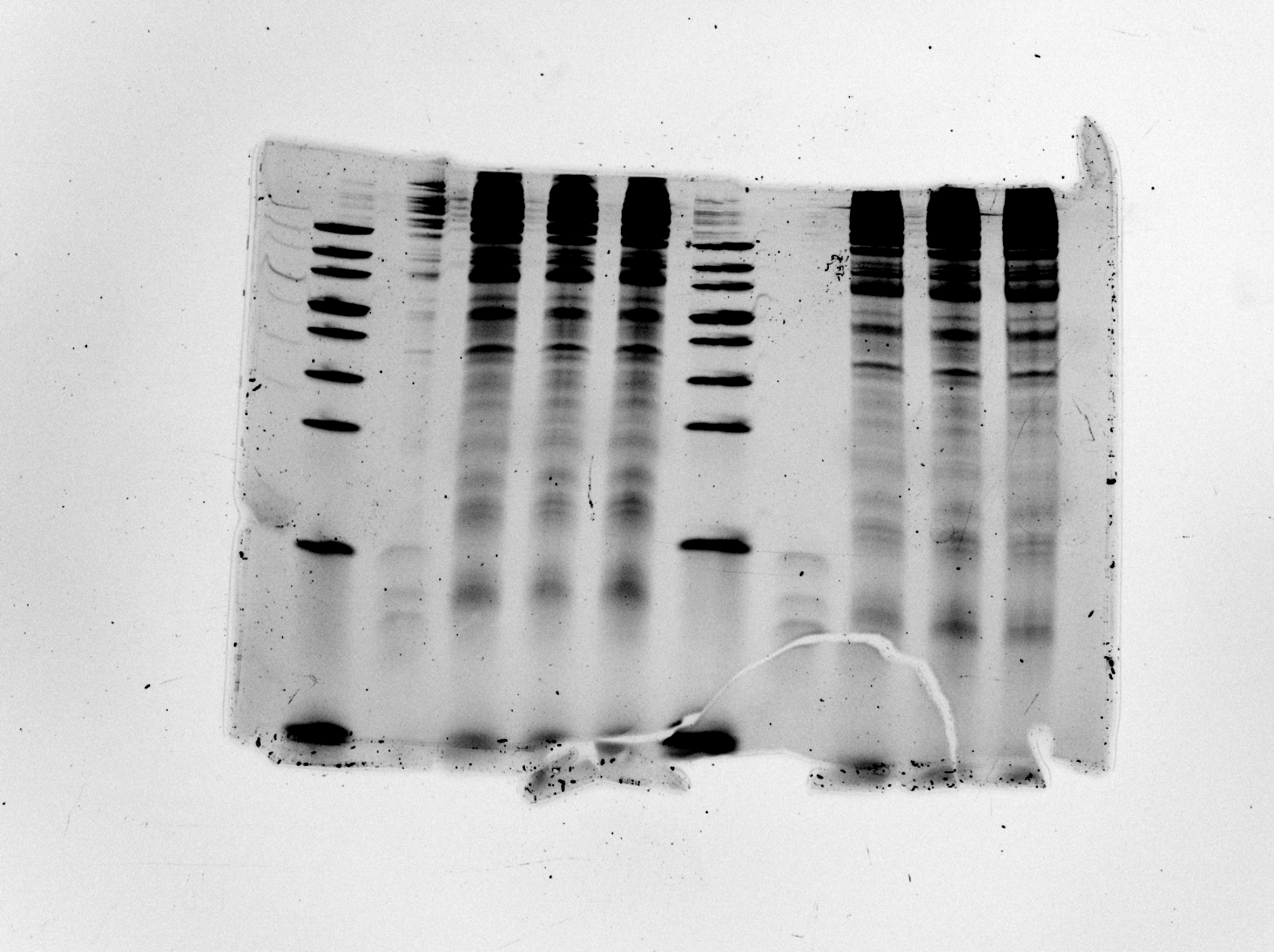

Supplement: Figure 6—figure supplement 1—source data 1. [file elife-78203-fig6-figsupp1-data1.zip › Figure 6_figure supplement 1_A.tif]

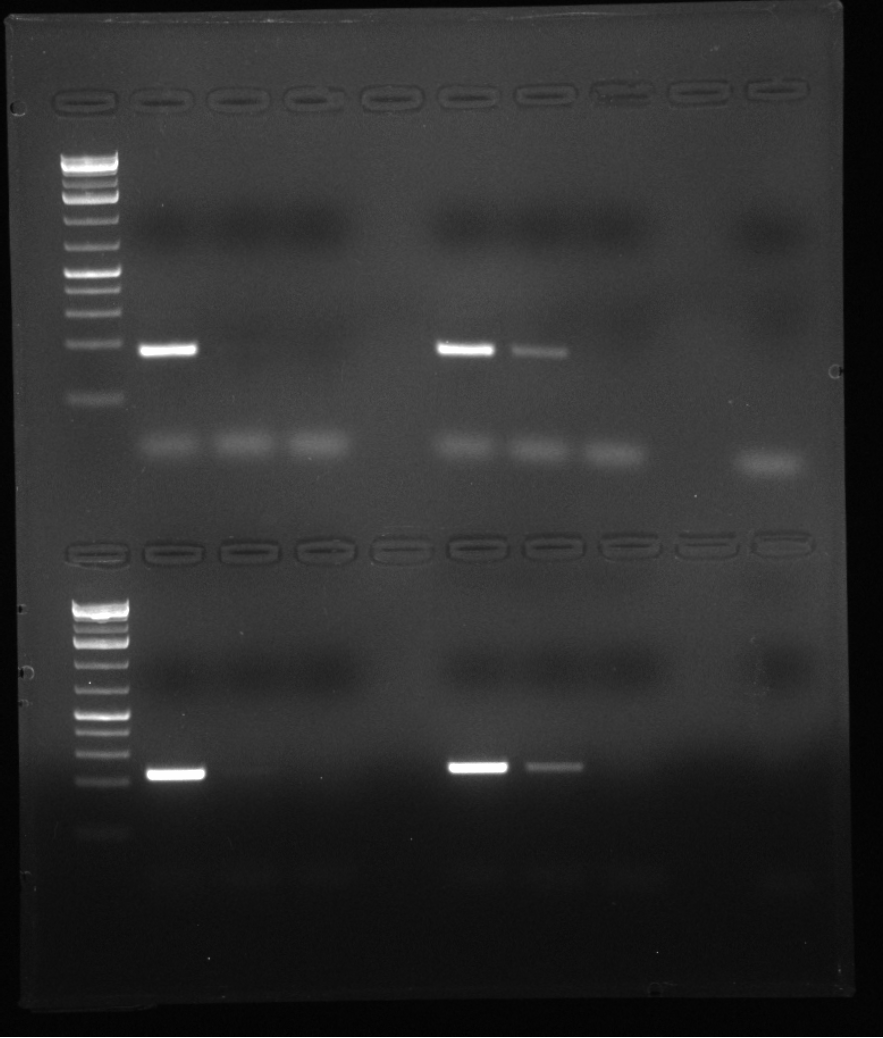

Supplement: Figure 6—figure supplement 2—source data 1. [file elife-78203-fig6-figsupp2-data1.zip › Figure 6_figure supplement 2_D_Setd3_Rcor2.jpg]

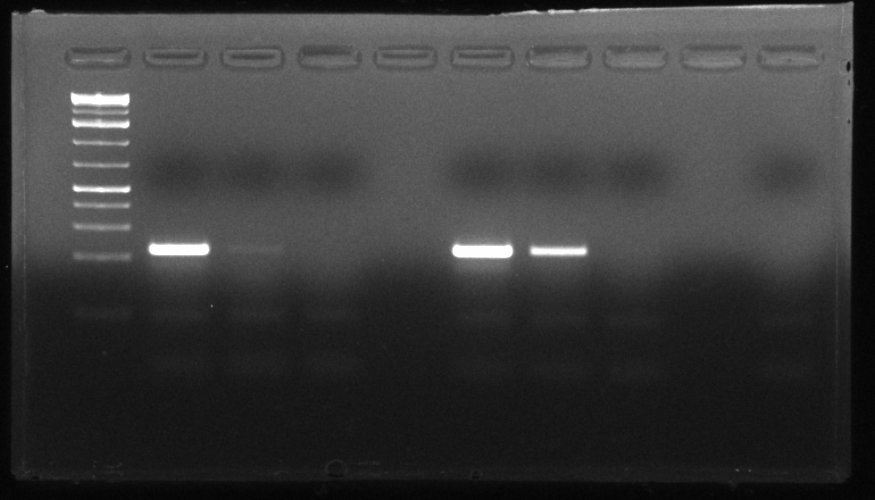

Supplement: Figure 6—figure supplement 2—source data 1. [file elife-78203-fig6-figsupp2-data1.zip › Figure 6_figure supplement 2_D_Topbp1.tif]
