# Supplementary material for: Aberrant cortical development is driven by impaired cell cycle and translational control in a DDX3X syndrome model
Source: eLife. 2022 Jun 28;11:e78203. doi: 10.7554/eLife.78203 (PMC9239684; doi:10.7554/eLife.78203)
Supplement: Transparent reporting form [file elife-78203-transrepform1.docx]

***eLife’s* transparent reporting form**

We encourage authors to provide detailed information *within their submission* to facilitate the interpretation and replication of experiments. Authors can upload supporting documentation to indicate the use of appropriate reporting guidelines for health-related research (see [EQUATOR Network](http://www.equator-network.org/%20)), life science research (see the [BioSharing Information Resource](https://biosharing.org/)), or the [ARRIVE guidelines](http://www.plosbiology.org/article/info:doi/10.1371/journal.pbio.1000412) for reporting work involving animal research. Where applicable, authors should refer to any relevant reporting standards documents in this form.

If you have any questions, please consult our Journal Policies and/or contact us: [editorial@elifesciences.org](mailto:editorial@elifesciences.org).

**Sample-size estimation**

- You should state whether an appropriate sample size was computed when the study was being designed
- You should state the statistical method of sample size computation and any required assumptions
- If no explicit power analysis was used, you should describe how you decided what sample (replicate) size (number) to use

Please outline where this information can be found within the submission (e.g., sections or figure legends), or explain why this information doesn’t apply to your submission:

We followed the ARRIVE guidelines and reported information regarding sample size, strain, use of littermates and both sexes, blinding of analyses, multiple investigators, etc in the methods. No explicit power analysis was performed; however, sample size was determined based on previous studies from our lab and others. For most of the analyses routinely performed in our lab, a sample size of 3-6 brains per condition is sufficient to detect a >20% change. For assays/analyses with greater variability, a larger sample size was used. We provided all of the detailed information on sample size, n, statistics, etc in Supplemental File 1. Some of this is in respective figure legends as well.

**Replicates**

- You should report how often each experiment was performed
- You should include a definition of biological versus technical replication
- The data obtained should be provided and sufficient information should be provided to indicate the number of independent biological and/or technical replicates
- If you encountered any outliers, you should describe how these were handled
- Criteria for exclusion/inclusion of data should be clearly stated
- High-throughput sequence data should be uploaded before submission, with a private link for reviewers provided (these are available from both GEO and ArrayExpress)

Please outline where this information can be found within the submission (e.g., sections or figure legends), or explain why this information doesn’t apply to your submission:

We have included this information in Supplemental File 1, which states the exact number of trials, sample size (including number of litters), and statistical test (with exact p-value) for each experiment. We also plotted individual data points for all graphs to represent the n (biological replicates) and color-coded by sex. Each n was calculated from at least 2-3 technical replicates. For the ribosome profiling/RNAseq experiment, one WT sample had to be excluded due to inefficient library prep, which is stated in the methods. Otherwise, no data were excluded and several null findings are included in the supplement.

**Statistical reporting**

- Statistical analysis methods should be described and justified
- Raw data should be presented in figures whenever informative to do so (typically when N per group is less than 10)
- For each experiment, you should identify the statistical tests used, exact values of N, definitions of center, methods of multiple test correction, and dispersion and precision measures (e.g., mean, median, SD, SEM, confidence intervals; and, for the major substantive results, a measure of effect size (e.g., Pearson's r, Cohen's d)
- Report exact p-values wherever possible alongside the summary statistics and 95% confidence intervals. These should be reported for all key questions and not only when the p-value is less than 0.05.

Please outline where this information can be found within the submission (e.g., sections or figure legends), or explain why this information doesn’t apply to your submission:

We have included details on statistical tests used, exact N’s, and exact p-values for each Figure panel in Supplemental File 1.

(For large datasets, or papers with a very large number of statistical tests, you may upload a single table file with tests, Ns, etc., with reference to sections in the manuscript.)

**Group allocation**

- Indicate how samples were allocated into experimental groups (in the case of clinical studies, please specify allocation to treatment method); if randomization was used, please also state if restricted randomization was applied
- Indicate if masking was used during group allocation, data collection and/or data analysis

Please outline where this information can be found within the submission (e.g., sections or figure legends), or explain why this information doesn’t apply to your submission:

We allocated samples into groups based upon genotype and sex. These are indicated in all figures by colors. All analyses were performed blind to genotype and in some cases by multiple reviewers.

**Additional data files (“source data”)**

- We encourage you to upload relevant additional data files, such as numerical data that are represented as a graph in a figure, or as a summary table
- Where provided, these should be in the most useful format, and they can be uploaded as “Source data” files linked to a main figure or table
- Include model definition files including the full list of parameters used
- Include code used for data analysis (e.g., R, MatLab)
- Avoid stating that data files are “available upon request”

Please indicate the figures or tables for which source data files have been provided:

We are including the original blots and ai source gel files for all figures.
